# Prognostic value of modified thrombelastometry for thromboembolic risk stratification in neurosurgical patients: protocol for a prospective, multicenter, observational study (TENS)

**DOI:** 10.1186/s41512-026-00244-9

**Published:** 2026-11-02

**Authors:** Hi-Jae Heiroth, Philipp Groene, Simon Schäfer, Matthias Längin, Kristina Fuest, Marion Hof, Jonas Alfitian, Tobias Brezina, Philipp Omuro, Saeed Torabi, Giusi Rueß-Paterno, Anne Göke, Simone Werschnik, Moritz Lenschow, Volker Neuschmelting, Anna Hagemeier, Tobias Kammerer

**Affiliations:** 1 Faculty of Medicine, Department of Anaesthesiology and Operative Intensive Care Medicine, University Hospital of Cologne, Cologne, Germany; 2 Department of Anaesthesiology, University Hospital of Munich (LMU), Munich, Germany; 3 Department of Anaesthesiology, Intensive Care and Emergency Medicine, DONAUISAR Hospital, Deggendorf, Germany; 4 Department of Anaesthesiology, Intensive Care, Emergency Medicine and Pain Therapy, Klinikum and Carl von Ossietzky University Oldenburg, Oldenburg, Germany; 5 Department of Anaesthesiology, University Hospital Klinikum rechts der Isar, TU München, Munich, Germany; 6 Faculty of Medicine, Department of General Neurosurgery, University Hospital of Cologne, Cologne, Germany; 7 Faculty of Medicine, Department of Neurosurgery, University Hospital of Berlin, Charité, Berlin, Germany; 8 Institute for Medical Statistics and Bioinformatics (IMSB), Faculty of Medicine and University Hospital, University of Cologne, Cologne, Germany

**Keywords:** Thromboelastometry, Thromboembolism, Neurosurgery

## Abstract

**Background and Objectives:**

Neurosurgical patients, particularly those with intracranial tumours such as glioma or meningioma, face a markedly elevated risk of venous thromboembolism (VTE), with reported rates of deep vein thrombosis between 15% and 70% depending on tumour entity and surgical extent. Current prophylactic strategies rely predominantly on pharmacological and mechanical measures and do not account for the individual complexity of coagulation dynamics. Modified thromboelastometry, employing a low-dose tissue plasminogen activator (TPA) test, enables functional assessment of both fibrinolytic capacity and prothrombotic states at the point of care. We hypothesize that preoperative and perioperative thromboelastometric parameters — in particular lysis time (LT) in the low-dose TPA test — are associated with the occurrence of thromboembolic events in neurosurgical patients.

**Methods:**

TENS is a prospective, multicenter, observational prognostic factor study targeting 350 evaluable participants (up to 369 enrolled to account for attrition) across four university hospitals in Cologne, Munich (two centres), and Oldenburg. Eligible patients are adults (≥ 18 years) scheduled for neurosurgical procedures involving intracranial tumour resection or with an anticipated operative duration of ≥ 4 h. Thromboelastometric measurements (EXTEM, INTEM, FIBTEM, low-dose TPA test) are performed at three defined time points: before surgery, at the end of surgery, and on postoperative day 1. The primary objective is to assess the prognostic association between lysis time (LT) in the low-dose TPA test and objectively imaging-confirmed venous thromboembolic events occurring within 30 days after surgery. Secondary endpoints comprise further thromboelastometric parameters, standard coagulation laboratory values, and clinical outcomes including length of hospital stay, duration of surgery, ICU stay, and days at home at day 30.

**Conclusions:**

Modified thromboelastometry has the potential to improve individualised risk stratification for thromboembolism in neurosurgical patients. If lysis time in the low-dose TPA test demonstrates prognostic value for thromboembolic events, this could inform the development of novel diagnostic and therapeutic algorithms for high-risk surgical populations.

**Trial registration, Date:**

Deutsches Register Klinischer Studien (DRKS), No. DRKS00034388, registered October 8, 2024. Anonymised participant-level data and the statistical analysis code will be made available upon reasonable request to the corresponding author following publication of the primary results, subject to applicable data protection regulations and the terms of the institutional data sharing agreement (see chapter The full study protocol will be publicly accessible via the German Clinical Trials Register (DRKS, No. DRKS00034388).Trial status: The study is currently in active recruitment. Recruitment was initiated at the coordinating centre in Cologne on 19 December 2024, with additional centres opening after completion of their local approval and site-initiation processes. As of March 2026, fewer than 50% of the target sample of 350 evaluable participants had been enrolled. Recruitment is expected to be completed by March 2027.

## Introduction

### Background

Neurosurgical patients are at substantially elevated risk for venous thromboembolism (VTE) [1], a complication that is not only potentially life-threatening but also prolongs hospitalisation, impairs quality of life, and delays return to a familiar social environment — consequences that are of particular relevance for patients with intracranial brain tumours. The incidence of deep vein thrombosis (DVT) among neurosurgical patients ranges from 15% to 45% [2, 3] and is strongly dependent on tumour pathology, surgical extent, and the patient’s clinical condition [4]. In patients with primary brain tumours such as glioma or meningioma, DVT rates of up to 70% have been reported, probably due to biological factors, specifically the release of procoagulant substances and fibrinolytic inhibitors from the tumour or surrounding tissue, which contribute to a subclinical hypercoagulable state and may be more important than traditional clinical risk factors [5–7]. Pulmonary embolism occurs in 1.4% to 5% of neurosurgical patients overall, rising to 3% to 10% in those classified as high risk [3, 5–8]. Recent reviews continue to identify patients with glioma, particularly glioblastoma, as a population at high risk of VTE and emphasise the competing risk of intracranial haemorrhage when preventive or therapeutic anticoagulation is considered [9–11].

Current preventive strategies focus predominantly on pharmacological and mechanical prophylaxis [12–17]. However, a critical diagnostic gap persists: the individual complexity of coagulation dynamics, clotting tendencies, and fibrinolytic capacity (the ability of the haemostatic system to break down fibrin-rich clots) is not captured by standard laboratory parameters such as platelet count, haemoglobin, International Normalized Ratio (INR), activated partial thromboplastin time (aPTT), or fibrinogen. These tests assess isolated aspects of the coagulation cascade and cannot reliably identify patients with altered functional coagulation despite values within the normal range. This limitation is clinically significant in neurosurgical patients, in whom the competing risks of thromboembolism and perioperative intracranial haemorrhage must be weighed simultaneously [18]. Importantly, uncritical substitution of coagulation factors such as factor XIII in the absence of functional testing may itself increase the risk of thromboembolism — a risk that standard laboratory parameters fail to detect [19].

Thromboelastometry addresses this diagnostic gap by providing a point-of-care assessment of the entire coagulation process, from clot initiation through clot formation and fibrinolysis (enzymatic breakdown of a fibrin clot) [20, 21]. The ROTEM delta^®^ system (Werfen, Munich) enables detection of both diffuse bleeding tendency and prothrombotic states, i.e. coagulation states favouring thrombosis [21]. Prior work from our group has demonstrated that the modified tissue plasminogen activator (TPA) test reliably detects residual antifibrinolytic activity, meaning inhibition of fibrin-clot breakdown, following tranexamic acid administration, underscoring its sensitivity to clinically relevant changes in fibrinolytic capacity [22, 23]. A particularly relevant extension of this technology is the low-dose TPA test, which quantifies fibrinolytic capacity within a blood sample and thereby enables systematic assessment of patients at elevated thrombotic risk. A recent systematic review of primary brain cancer reported evidence of increased secondary haemostatic activity and impaired fibrinolysis, including prolonged clot lysis time, supporting the biological rationale for this approach [24]. Despite this mechanistic rationale, the prognostic value of modified thromboelastometry — and specifically the low-dose TPA test — has not yet been systematically evaluated in neurosurgical patients. This study addresses that gap.

### Study objectives and hypotheses

The primary objective of this study is to determine whether modified thromboelastometry, specifically the lysis time (LT) measured in the low-dose TPA test, is associated with the occurrence of thromboembolic events in neurosurgical patients with intracranial tumours or surgical procedures of ≥ 4 h duration.

Secondary objectives are to investigate: (1) whether preoperative pathological thromboelastometric values are associated with postoperative thromboembolic complications; (2) whether perioperative changes in thromboelastometric parameters — from the preoperative baseline to postoperative day 1 — are predictive of thrombotic events; (3) whether pathological values in the low-dose TPA test identify patients at elevated risk for perioperative haemorrhage; and (4) whether demographic and/or perioperative variables (including age, BMI, and pre-existing medication, fluids, surgery time) in combination with thromboelastometric parameters can be used in multivariable analysis to identify independent predictors of postoperative thromboembolic events and (5) how these events affect long-term outcome.

The findings of this study are intended to inform the development of novel diagnostic and therapeutic algorithms for neurosurgical patients at high risk for thromboembolism or perioperative haemorrhage.

## Methods: Participants, interventions and outcomes

### Study design

TENS is designed as a prospective, multicenter, observational prognostic factor study conducted across four university hospitals in Germany: University Hospital of Cologne, Ludwig-Maximilians-Universität (LMU) Munich, Technical University of Munich, and University Hospital of Oldenburg. The coordinating centre is the Department of Anaesthesiology and Intensive Care Medicine, University Hospital of Cologne, Faculty of Medicine, University of Cologne. Each centre is responsible for screening, enrolment, local data entry into the electronic case report form (eCRF) system (Castor EDC), and on-site thromboelastometric measurements using standardised equipment and reagents. Central data monitoring and statistical analysis are coordinated by the principal investigator and the Institute for Medical Statistics and Bioinformatics at the University of Cologne. The study follows a longitudinal design with repeated measurements at three predefined time points: (1) before surgery, immediately following anaesthesia induction; (2) at the end of surgery, upon completion of the main surgical procedure; and (3) on postoperative day 1. No randomisation or experimental intervention is performed. The study evaluates the prognostic association between thromboelastometric parameters — particularly the low-dose TPA test — and objectively confirmed VTE occurring within 30 days after surgery. Ethical approvals are detailed in the Declarations section. This protocol manuscript has been aligned with the Standardized Protocol Items Recommendations for Observational Studies (SPIROS), and reporting of the subsequent prognostic factor study will follow the Reporting Recommendations for Tumor Marker Prognostic Studies (REMARK), adapted to this non-tumour-marker prognostic factor setting [25, 26].

### Participants

#### Eligibility criteria

Patients are eligible for inclusion if they meet all of the following criteria: age ≥ 18 years; scheduled for an elective neurosurgical procedure at one of the four participating centres; and either (a) diagnosed with an intracranial tumour (including but not limited to glioma, meningioma, or brain metastasis) or (b) scheduled for a neurosurgical procedure with an anticipated operative duration of ≥ 4 h; and provision of written informed consent prior to enrolment.

The only exclusion criterion is refusal to participate or ineligibility for informed consent. Patients with pre-existing haemostatic disorders, known coagulopathies, or current anticoagulant therapy are intentionally not excluded in order to preserve the clinical representativeness of this high-risk neurosurgical cohort. These characteristics are recorded prospectively and will be considered in unadjusted and adjusted analyses; additional sensitivity analyses will be performed where event numbers permit.

Informed consent will be obtained by trained anaesthesiologists and surgeons at each participating centre. Eligible patients will be identified during the preoperative anaesthesia assessment and will receive written study information at least 24 h prior to the scheduled procedure, allowing adequate time for consideration. Written informed consent will be obtained before any study-related procedures are initiated and prior to enrolment. The informed consent process strictly adheres to the principles of the Declaration of Helsinki and applicable German regulatory requirements.

#### Additional consent provisions for collection and use of participant data and biological specimens

The informed consent form explicitly covers the collection, pseudonymised storage, and use of participant data and biological specimens for this study. Participants are additionally asked to consent to the long-term storage of biological specimens at -80 °C for up to 10 years, and to the potential use of pseudonymised data for future research on coagulation and perioperative medicine, in accordance with applicable data protection regulations. Participants may withdraw consent at any time without disadvantage to their medical care.

#### Confidentiality

All participant data are handled in strict accordance with the General Data Protection Regulation (GDPR) and the German Federal Data Protection Act (Bundesdatenschutzgesetz, BDSG). Each enrolled participant is assigned a unique pseudonymous study identification number; no directly identifying information is entered into the eCRF. The linking key connecting the study identification number to the participant’s identity is stored separately in a password-protected, access-restricted file at the coordinating centre and is accessible solely to the principal investigator and, within their respective centre, to the designated site investigator. Authorised regulatory authorities and ethics committees retain the right to access source data for monitoring and audit purposes, in accordance with applicable GCP guidelines. All study data will be retained for a minimum of 10 years following study completion.

#### Plans for collection, laboratory evaluation and storage of biological specimens for genetic or molecular analysis in this trial/future use

Blood samples for plasmatic coagulation parameters are collected at each participating centre at the defined study time points and are processed locally according to standardised operating procedures provided by the coordinating centre. Aliquots are stored at − 80 °C at the local site until shipment to the coordinating centre in Cologne, where all central laboratory analyses are performed. Samples are transported on dry ice under temperature-controlled conditions in accordance with applicable biosafety and transport regulations. Following analysis, all biological specimens are destroyed in accordance with institutional waste disposal regulations; no long-term biobanking of study material is planned beyond the period required for analysis. In accordance with the informed consent obtained from participants, pseudonymised samples may be used exclusively for research questions directly related to the objectives of the TENS study. All samples and associated data are retained for a minimum of 10 years following study completion, in accordance with GCP guidelines and applicable regulatory requirements.

### Data collection and management

#### Plans for assessment and collection of outcomes

All study data are collected by designated staff at each participating centre and entered prospectively into a validated eCRF using the Castor EDC platform (Castor, Amsterdam, Netherlands), which is accessible exclusively to authorised study personnel. Clinical outcome data, including thromboembolic events and secondary clinical endpoints, are extracted from the electronic patient record at each centre. Thromboelastometric raw data are recorded directly at the point of care and transferred to the eCRF by the performing physician or postgraduate researcher. All data are archived in pseudonymised form in accordance with Good Clinical Practice (GCP) guidelines and applicable data protection regulations. Long-term data preservation is ensured for a minimum of 10 years following study completion, with accessibility for future reuse by authorised third parties subject to individual privacy and data protection requirements.

#### Plans to promote participant retention and complete follow-up

Given the non-interventional nature of the study and the fact that all blood samples are obtained from already indwelling catheters without additional venous punctures, the burden on individual participants is minimal and a dropout rate of less than 5% is anticipated. Participant retention is actively supported through a structured telephone follow-up at day 30 after surgery, conducted by study personnel at each centre. If the participant cannot be reached by telephone, at least two further contact attempts are made on separate days before the participant is classified as lost to follow-up. Participants who withdraw consent are not replaced; their data collected up to the point of withdrawal are retained for analysis, subject to the consent form.

#### Data management

Participant data are entered into the eCRF under a unique pseudonymous study identification number. Access to the eCRF is role-based and restricted to authorised study personnel at each participating centre; access rights are assigned and managed centrally by the principal investigator. Storage of the linking key and protection of directly identifying information are described above under Confidentiality.

Data quality is ensured through built-in validation rules within the eCRF, including range checks, mandatory field definitions, and automated plausibility checks. Any data queries are resolved through documented communication between the coordinating centre and the responsible site investigator. Source data verification is performed regularly by the coordinating centre to ensure consistency between eCRF entries and source documents.

The relevance of biological sex is explicitly considered in the study design. Sex is recorded as a demographic variable for all participants, will be included as a candidate covariate in the adjusted analysis, and will be explored in subgroup analyses of thromboelastometric parameters and thromboembolic risk. Gender identity is not collected as a study variable.

### Test methods

#### Rotational thromboelastometry (ROTEM) and Low-Dose tPA-Test

Viscoelastic coagulation testing will be performed using the ROTEM delta system (Tem Innovations GmbH, Munich, Germany) according to a standardised operating procedure across all participating centres. Whole blood will be collected in citrate tubes and analysed within the recommended time window. The standard assays comprise EXTEM, INTEM, and FIBTEM, assessing the extrinsic, intrinsic, and fibrinogen-dependent components of clot formation, respectively. In addition, a modified low-dose TPA assay (EXTEM L) will be performed to evaluate fibrinolytic capacity. For this purpose, recombinant TPA (alteplase) will be prepared as a stock solution (1 mg/mL), further diluted to an intermediate concentration of 0.1 mg/mL, and finally adjusted to a working concentration of 0.01 mg/mL. For each low-dose TPA measurement, 10 µL of the diluted TPA solution will be added to 1 mL citrated whole blood, followed by a standardised incubation period of 60 s before analysis. Samples will then be processed in parallel across four ROTEM channels (INTEM, EXTEM, FIBTEM, and EXTEM-TPA). Measurements will be performed according to the manufacturer’s instructions using automated pipetting steps (STARTEM, assay-specific reagents, and air-gap technique). All assays will run at 37 °C. Recorded parameters comprise clotting time (CT), clot formation time (CFT), maximum clot firmness (MCF), lysis index (LI30/45/60), and lysis time (LT). The primary parameter of interest for the low-dose TPA assay is LT, defined as the time from clot formation to a predefined degree of clot breakdown and interpreted as a measure of fibrinolytic resistance. To ensure reproducibility, all devices will be calibrated regularly, personnel will undergo structured training, and measurements will be documented and stored digitally immediately after completion. Plasma samples will additionally be processed by centrifugation (2000 g, 10 min, 4 °C) and stored at − 80 °C for subsequent biomarker analyses. The three ROTEM time points were selected to characterise baseline, immediate postsurgical, and early postoperative changes for prognostic assessment; later ROTEM measurements are not planned because the study is designed to evaluate early perioperative prognostic markers rather than serial coagulation monitoring throughout the 30-day follow-up.

### Explanation for the choice of comparators

TENS is a prospective observational diagnostic study without any experimental intervention or comparator arm. Thromboelastometric measurements are performed as an adjunct to routine perioperative care and do not influence clinical decision-making or treatment. The study therefore does not involve allocating participants to intervention or comparator groups.

### Participant follow-up and outcome ascertainment

Participants are followed from the day of surgery through postoperative day 30. During the index hospitalisation, VTE surveillance is symptom-driven as part of routine clinical care. Clinical findings suggestive of DVT or pulmonary embolism, or a D-dimer result that raises clinical suspicion, prompt clinically indicated diagnostic imaging. For classification of the primary endpoint, imaging confirmation is mandatory: DVT must be confirmed by compression ultrasonography and pulmonary embolism by computed tomography pulmonary angiography. Clinical signs or D-dimer elevation alone are insufficient for primary endpoint classification. At the structured day-30 telephone follow-up, participants are asked about interval thromboembolic diagnoses, bleeding complications, re-hospitalisation, re-operations or interventions, and days at home. Any reported thromboembolic event must be verified from the corresponding medical record and imaging report before it is classified as a primary endpoint event. Systematic imaging screening of asymptomatic participants is not part of the approved observational protocol.

### Analysis

#### Primary endpoint

The primary endpoint is the occurrence of an objectively confirmed venous thromboembolic event from surgery through postoperative day 30. Events comprise DVT confirmed by compression ultrasonography or pulmonary embolism confirmed by computed tomography pulmonary angiography. Clinical signs and D-dimer elevation may trigger diagnostic evaluation but are not sufficient for primary endpoint classification.

#### Secondary endpoints — thromboelastometric parameters

Secondary thromboelastometric endpoints are assessed using the ROTEM delta^®^ system (Werfen, Munich) at all three predefined time points (before surgery, at the end of surgery, and on postoperative day 1). The complete set of recorded parameters comprises: clotting time (CT), clot formation time (CFT), alpha angle, amplitude at 5, 10, 15, 20, 25, and 30 min (A5, A10, A15, A20, A25, A30), maximum clot firmness (MCF), time to maximum clot firmness (MCF-t), lysis index at 30, 45, and 60 min (LI30, LI45, LI60), maximum lysis (ML), clot formation rate (CFR), lysis onset time (LOT), clot lysis rate (CLR), area-under-the-curve-derived parameters at 5, 10, 20, 25, and 30 min (AR5, AR10, AR20, AR25, AR30), maximum clot elasticity (MCE), actual clot firmness (ACF), thrombodynamic potential index (TPI), maximum clot velocity (MaxV), time to maximum clot velocity (MaxV-t), and area under the aggregation curve (AUC). All thromboelastometric measurements will be performed and analysed by trained study personnel.

#### Secondary endpoints — plasmatic coagulation parameters

Plasmatic coagulation parameters are measured before surgery, at the end of surgery, and on postoperative day 1. The predefined panel comprises aPTT, fibrinogen (Clauss method), plasminogen activator inhibitor antigen (PAI-AG), active plasminogen activator inhibitor (aPAI), TPA, von Willebrand factor antigen (vWF-AG), von Willebrand factor activity, and factor XIII.

#### Secondary endpoints — standard laboratory parameters

Standard laboratory parameters are recorded at screening and, where available as part of routine clinical care, at subsequent time points. The predefined parameters comprise red blood cell count, haemoglobin, haematocrit, white blood cell count, and platelet count.

#### Secondary endpoints — clinical outcomes

Clinical secondary endpoints assessed during the 30-day follow-up comprise: clinical signs suggestive of DVT (Payr, Homans, and Meyer signs) or pulmonary embolism (dyspnoea, chest pain, cough, tachycardia, cyanosis); D-dimer levels and clinically indicated diagnostic workup; length of hospital stay (LOS); length of ICU stay (LOICUS); days at home at day 30 (DAH-30); duration of mechanical ventilation (hours); need for re-operation or re-intervention; transfusion requirements and blood product utilisation; administration of tranexamic acid; duration of surgery; and perioperative fluid management.

#### Sample size

Sample size calculation was performed a priori using PASS 2022 Power Analysis (NCSS, LLC., Kaysville, Utah, USA). The primary prognostic assessment is based on discrimination of LT in the low-dose TPA test for the occurrence of a 30-day thromboembolic event, quantified by the area under the receiver operating characteristic curve (AUC-ROC). In the absence of prior neurosurgical data for this modified assay, an alternative-hypothesis AUC of 0.725 was selected prospectively as the target level of discrimination considered sufficient to justify subsequent clinical validation; it represents an absolute improvement of 0.225 over chance discrimination (AUC = 0.5) and was not derived from pilot data. Based on a one-sided z-test with α = 0.025 and 80% power, a minimum of 14 participants with confirmed thromboembolic events and 336 participants without events are required, yielding an achieved power of 82% under these assumptions. The ratio of standard deviations between negative and positive groups is assumed to be 1. Allowing for 5% attrition, up to 369 participants will be enrolled to obtain 350 evaluable participants (14 event-positive and 336 event-negative). Recruitment is distributed across four centres over a planned 12- to 18-month inclusion period without a fixed monthly quota per centre because site activation and recruitment rates may differ. If the true AUC is lower than 0.725, the study will have correspondingly lower power; this limitation is acknowledged explicitly in the Discussion.

#### Statistical methods for primary and secondary outcomes

All statistical analyses will be performed using R Version 4.5.3 or higher (R Foundation for Statistical Computing, Vienna, Austria) and IBM SPSS Statistics Version 31 (IBM Corp., Armonk, NY, USA). Continuous variables will be reported as mean ± standard deviation (SD) and median with interquartile range (IQR). Distributional assumptions will be assessed primarily by graphical inspection of histograms and Q-Q plots together with descriptive summaries rather than by reliance on a formal normality test alone. Parametric methods (Student’s t-test or ANOVA) will be used when their assumptions are not clearly implausible; otherwise, Mann-Whitney U or Kruskal-Wallis tests will be used as appropriate. Categorical variables will be reported as absolute and relative (%) frequencies and compared using the chi-squared test or Fisher’s exact test, as appropriate. A two-sided significance level of α = 0.05 will be applied for inferential analyses unless otherwise specified for the prespecified primary AUC analysis.

For the primary analysis, LT in the low-dose TPA test will be analysed as a continuous prognostic factor; no clinical cut-off is prespecified. The primary discrimination measure will be the unadjusted ROC-AUC for LT alone, with a 95% confidence interval estimated using 2,000 stratified non-parametric bootstrap resamples. An exploratory data-derived LT threshold will be estimated using the Youden index and will be reported explicitly as exploratory rather than as a validated clinical cut-off; bootstrap resampling will be used to assess its internal stability and optimism. The prognostic association between LT and 30-day VTE will first be quantified in an univariable binary logistic regression model and then in an exploratory adjusted model. Candidate covariates defined a priori are biological sex, pre-existing haemostatic disorders, current anticoagulant therapy at the time of surgery (warfarin, direct oral anticoagulants, or low-molecular-weight heparin), BMI, tumour entity, and surgical duration. Both unadjusted and adjusted odds ratios (ORs) with 95% confidence intervals will be reported. Because the expected number of VTE events is limited, the adjusted analysis will be considered exploratory; model complexity will be constrained by the observed event count, and penalised logistic regression will be used if conventional maximum-likelihood estimation would be unstable. Internal bootstrap validation will be used to quantify optimism in multivariable model performance.

For secondary thromboelastometric endpoints, changes across the three measurement time points (before surgery [Visit 1], at the end of surgery [Visit 2], and on postoperative day 1 [Visit 3]) will be analysed using linear mixed models for repeated measures (LMMRM). Models will include time, age, and sex as fixed effects and patient ID as a random intercept to account for within-participant correlation. Results will be presented as estimated marginal means (EMMs) with corresponding 95% confidence intervals for each time point.

#### Methods for additional analyses

Prespecified exploratory subgroup analyses will assess potential effect modification by tumour entity (glioblastoma, meningioma, astrocytoma, and other), surgical duration (≥ 4 h versus shorter procedures in patients with intracranial tumours), current therapeutic anticoagulation, and known haemostatic disorders, where event numbers permit. Given the expected small number of VTE events, subgroup findings will be interpreted cautiously and considered hypothesis-generating.

#### Methods in analysis to handle protocol non-adherence and any statistical methods to handle missing data

The primary analysis population will comprise all enrolled participants with ascertainable 30-day VTE status. The primary unadjusted ROC analysis requires an observed baseline LT value; participants without an analysable baseline LT will therefore be excluded from the complete-case primary ROC analysis. A sensitivity analysis will additionally exclude participants with major deviations from the predefined thromboelastometry measurement schedule; this is termed the measurement-adherent sensitivity population rather than a per-protocol population because no treatment is assigned. Missing predictor and covariate data, including incomplete LT measurements where imputation is considered clinically and statistically plausible, will be addressed in a multiple-imputation sensitivity analysis using chained equations (MICE) under a missing-at-random assumption [27]. Imputation will be performed jointly across centres, with study centre included as a predictor rather than by fitting separate centre-specific imputation models. The imputation model will include the 30-day VTE outcome, LT, all variables in the planned analysis models, and relevant auxiliary variables. The primary VTE outcome itself will not be imputed. The number of imputations will be at least 10 and will be increased approximately in line with the proportion of participants with incomplete data. Results from the imputed datasets will be combined using Rubin’s rules and compared with complete-case analyses.

#### Plans to give access to the full protocol, participant level-data and statistical code

The full study protocol will be publicly available via the German Clinical Trials Register (DRKS, No. DRKS00034388). To ensure participant privacy, data will be de-identified prior to sharing by removing all direct identifiers (e.g., names, specific dates, and subject IDs linked to hospital records). Quasi-identifiers, such as age and body mass index, will be generalized into categories where necessary to prevent re-identification. The anonymization process will be conducted in accordance with the GDPR standards and verified using internal data privacy audits before release.

**Table 1:** Participant timeline and assessment schedule. Visit 1 = baseline assessment in the operating room immediately following anaesthesia induction; Visit 2 = assessment at the end of surgery in the operating room or on the ICU; Visit 3 = assessment on postoperative day 1; Visit 4 = optional assessment on postoperative days 5–7, performed exclusively in patients still hospitalised at that time point; Follow-up = structured telephone interview at day 30 after surgery to assess clinical outcomes including thromboembolic events and days at home (DAH-30). (√) = if available as part of routine clinical care

	Screening before surgery	Visit 1 in OR before surgery	Visit 2 end of surgery	Visit 3 day 1 after surgery	Visit 4 (optional) day 5–7 after surgery	Follow-up 1 month postoperatively
Informed consent	√					
In-/exclusion criteria	√					
Medical history of patient	√					
Thrombelastometry		√	√	√		
Standard laboratory tests	√	(√)	(√)	(√)	(√)	
Plasmatic coagulation variables		√	√	√	(√)	
Complete Case Report Forms (CRFs)			√		√	√
Phonecall						√

### Participants

#### Participant timeline

The participant timeline is summarised in Table 1. The total study period per participant spans 30 days from the date of surgery. Following an initial screening visit before surgery, participants are assessed at three defined thromboelastometry time points: before surgery (Visit 1, immediately following anaesthesia induction), at the end of surgery (Visit 2, upon completion of the main surgical procedure), and on postoperative day 1 (Visit 3). An additional optional clinical assessment is performed on postoperative days 5–7 (Visit 4) in participants who remain hospitalized. A structured telephone follow-up is conducted on day 30 to ascertain interval clinical outcomes, including reported VTE diagnoses, bleeding complications, re-hospitalisation, and days at home within 30 days (DAH-30); suspected or reported VTE events are verified against medical records and imaging reports before primary endpoint classification.

Eligible patients are identified through daily screening of the surgical schedule at each participating centre by designated study physicians. Patients meeting the inclusion criteria are approached during the preoperative anaesthesia assessment, and informed consent is obtained as described above under Informed consent. Recruitment is pooled across the four participating centres with a planned inclusion period of 12–18 months and a target of up to 369 enrolled participants to obtain 350 evaluable participants. No fixed monthly recruitment quota is imposed on individual centres because site activation and recruitment rates may differ. Recruitment feasibility is supported by the large neurosurgical case volume across the participating centres and the low additional study burden, as study blood sampling is integrated into perioperative blood collection from indwelling catheters.

### Oversight and monitoring

#### Composition of the coordinating centre and trial steering committee

The coordinating centre is the Department of Anaesthesiology and Intensive Care Medicine, University Hospital of Cologne. The principal investigator at the coordinating centre is responsible for overall study coordination, protocol adherence, central data monitoring, and communication with the ethics committees and the trial registry. Each participating centre designates a site investigator who is responsible for local patient screening, enrolment, data entry, and sample management. The trial steering committee comprises the principal investigator and all site investigators. Regular steering committee meetings are held at least quarterly, either in person or via video conference, and are chaired by the principal investigator. Meeting minutes are documented and archived at the coordinating centre. Ad hoc meetings may be convened at the principal investigator´s discretion in the event of protocol deviations, safety concerns, or operational issues requiring immediate attention.

#### Composition of the data monitoring committee, its role and reporting structure

A formal data monitoring committee (DMC) is not established for this study. Given the non-interventional, observational nature of TENS, there is no experimental intervention that could expose participants to treatment-related harm, and no interim efficacy analyses are planned. The study risk profile is therefore considered low and does not warrant independent data monitoring. Data quality and protocol adherence are monitored by the principal investigator at the coordinating centre through regular source data verification and eCRF review, as described above.

#### Frequency and plans for auditing trial conduct

Formal external auditing is not planned, given the non-interventional nature of the study and the absence of an investigational medicinal product or device. Internal quality assurance is performed by the principal investigator through regular review of eCRF data, source data verification at participating centres, and quarterly steering committee meetings. In the event of a suspected protocol deviation or data integrity concern, the principal investigator will initiate an internal review and document the findings and corrective actions taken.

#### Plans for communicating important protocol amendments to relevant parties (e.g. trial participants, ethical committees)

Ethics approvals for all participating centres are reported in the Declarations section. Any substantial protocol amendments — defined as modifications that may affect participant safety, scientific validity, or participants’ rights — will be submitted to the responsible ethics committees and will not be implemented before approval. Non-substantial administrative changes will be documented internally and communicated to all site investigators. Protocol amendments will be updated in the German Clinical Trials Register (DRKS, No. DRKS00034388) in a timely manner. Participants will be informed of amendments that may affect their willingness to continue participation, and re-consent will be obtained where required.

#### Dissemination plans

Results of the TENS study will be disseminated through publication in a peer-reviewed, open-access journal following completion of the primary outcome analysis. Authorship will be determined in accordance with the criteria of the International Committee of Medical Journal Editors (ICMJE). Findings will also be presented at national and international scientific meetings relevant to the fields of anaesthesiology, haemostaseology, and neurosurgery, including but not limited to the annual congresses of the Deutsche Gesellschaft für Anästhesiologie und Intensivmedizin (DGAI) and the European Society of Anaesthesiology and Intensive Care (ESAIC). A summary of results will be made available to participants upon request. Results will be reported regardless of direction or magnitude of effect, in accordance with the principles of responsible research reporting.

## Discussion

The TENS study addresses a clinically relevant and currently unresolved diagnostic gap in the perioperative management of neurosurgical patients. While the elevated thromboembolic risk in this patient population is well established [1–7], existing prophylactic strategies rely predominantly on pharmacological and mechanical measures and do not incorporate a systematic functional assessment of individual coagulation dynamics. Standard laboratory parameters reflect only isolated components of the coagulation cascade and are insufficient to identify patients whose functional coagulation state places them at elevated risk for thromboembolism or perioperative haemorrhage [14, 15]. Modified thromboelastometry, and the low-dose TPA test in particular, offers a point-of-care approach to functional coagulation assessment that has not yet been systematically evaluated in the neurosurgical setting [16, 17]. The present study is, to our knowledge, the first prospective multicenter investigation to evaluate the diagnostic value of this approach specifically in neurosurgical patients with intracranial tumours or prolonged operative duration.

Several methodological strengths support the study’s validity. The prospective, multicenter design enhances the generalisability of findings across different institutional settings. Standardised equipment and reagents are provided centrally to all participating centres, minimising inter-site variability in thromboelastometric measurements. The non-interventional nature of the study, with all blood samples obtained from already indwelling catheters, results in minimal participant burden and is expected to support high consent and retention rates. The longitudinal measurement design at three predefined time points enables assessment of dynamic perioperative changes in coagulation, rather than a single static measurement.

The study also has inherent limitations that must be acknowledged. As an observational prognostic factor study, it cannot establish causality between thromboelastometric parameters and thromboembolic events. The number of participants expected to develop confirmed VTE is relatively small by design, limiting precision of adjusted and subgroup analyses. The sample size calculation assumes an AUC of 0.725; because this value is an a priori design target rather than an estimate derived from prior low-dose TPA data in neurosurgical patients, the study may be underpowered if the true discrimination is lower. VTE surveillance is symptom-driven and no systematic imaging screening of asymptomatic participants is planned; consequently, asymptomatic events may be missed and detection bias cannot be excluded. In addition, ROTEM measurements are limited to the preoperative period, the end of surgery, and postoperative day 1. These time points characterise the early perioperative trajectory but do not capture later coagulation changes between postoperative day 1 and a subsequent VTE event. The study is not powered to detect differences between individual tumour entities or surgical subgroups, and such findings will be exploratory. Finally, perioperative confounders such as mild hypothermia are known to influence thromboelastometric parameters [28] and will be recorded for appropriate consideration in the analyses.

Should the low-dose TPA test prove to be a reliable predictor of thromboembolic events in neurosurgical patients, these findings could inform the development of individualised, thromboelastometry-guided diagnostic and prophylactic algorithms for this high-risk population, with potential implications for patient safety, length of hospital stay, and healthcare resource utilisation. Should no significant association be demonstrated, this would itself constitute an important contribution to the evidence base, clarifying the boundaries of thromboelastometry as a diagnostic tool in the neurosurgical context and guiding the design of future investigations.

## Data Availability

No datasets were generated or analysed during the current study.
